# Free Convection in a Parallelogrammic Porous Cavity Filled with a Nanofluid Using Tiwari and Das’ Nanofluid Model

**DOI:** 10.1371/journal.pone.0126486

**Published:** 2015-05-19

**Authors:** Mohammad Ghalambaz, Mikhail A. Sheremet, Ioan Pop

**Affiliations:** 1 Department of Mechanical Engineering, Dezful Branch, Islamic Azad University, Dezful, Iran; 2 Department of Theoretical Mechanics, Faculty of Mechanics and Mathematics, Tomsk State University, Tomsk, Russia; 3 Institute of Power Engineering, Tomsk Polytechnic University, Tomsk, Russia; 4 Department of Applied Mathematics, Babeş-Bolyai University, Cluj-Napoca, Romania; North China Electric Power University, CHINA

## Abstract

The free convection heat transfer of Cu-water nanofluids in a parallelogrammic enclosure filled with porous media is numerically analyzed. The bottom and top of the enclosure are insulated while the sidewalls are subject to limited temperature difference. The Darcy flow and the Tiwari and Das’ nanofluid models are considered. The governing dimensionless partial differential equations are numerically solved using a finite difference code. The results are reported for isotherms and streamlines as well as Nusselt number as a function of the volume fraction of nanoparticles, porosity, types of the porous matrix, inclination angle, aspect ratio and different Rayleigh numbers. It is found that the presence of the nanoparticles inside the enclosure deteriorates the heat transfer rate, which is caused due to the increase of dynamic viscosity by the presence of nanoparticles. Therefore, in applications in which the nanofluids are used for their advantages, such as enhanced dielectric properties or antibacterial properties, more caution for the heat transfer design of the enclosure is necessary.

## Introduction

The convective heat transfer in enclosures is important, as it does not need an external power source for inducing convective heat transfer, so that in this type of convective heat transfer, there is no need of any electrical supplies and electronic regulation. The natural convection process, also excludes the risks of mechanical malfunction existing for systems owing due to forced convection flow. The absence of external power supplies systems reduces the cost, sound and the magnetic noise in natural convection process. These are only some of the advantages of the natural convection flows, which continuously increases the interest of industrial sections and researchers to resort to this heat transfer phenomenon. In addition, the natural convection phenomena are especially important for equipment located in limited volumes (or enclosures), which frequently are encountered in many industrial applications and situation. Baïri et al. [1] have reported an excellent review on the natural convection in enclosures for engineering applications. In many cases, the enclosures are filled with a porous medium, which is saturated by a fluid. Convection in porous media has many applications in several sections of industries such as cooling of electronic devices, buildings, solar collectors, geothermal energy, fuel cells, food, etc. [2–6].

The nanofluids are a new type of engineered fluids, which contain well-dispersed nanoparticles [7, 8]. The presence of the nanoparticles in the basic or working fluid affects substantially its properties. For example, the diamond nanoparticles [9] or ceramic nanoparticles [10] are used as an additive to the mineral oil for electrical power transformers and other oil-cooled electrical equipment to enhance the dielectric properties of the oil. The presence of nanoparticles in the base fluid has also found applications in solar collectors, which can transform the resulting nanofluid to a medium for direct absorption of the sun light in the solar collectors [11–13]. The ZnO and TiO_2_ nanoparticles have found medical applications as they show sustainable antibacterial activities under specific conditions [14, 15]. The presence of nanoparticles also modifies the thermo-physical properties of the host fluid. Experiments show that the thermal conductivity, density, and viscosity of the resulting nanofluid are higher than that of the base fluid [16]. Therefore, the convective heat transfer of nanofluid can be affected by the presence of nanoparticles in the base fluid, and is essential in applications. Several researchers have numerically and experimentally studied nanofluids inside cavities including thermal conductivity [17] and solid volume fraction of the nanoparticles [18, 19].

Costa [20] studied the natural convection flow and heat transfer in a parallelogrammic enclosures filled with a fluid-saturated porous media using Darcy model. The top and bottom walls were adiabatic and the sidewalls were subject to temperature difference. Costa analyzed the effect of different aspect ratios and inclination angles on the average Nusselt number. The results were reported for Rayleigh number in the range of 10 to 100. It is found that the increase of the aspect ratio (*A* = *H*/*L*, where *H* is the height and *L* is the length of the enclosure) increases the average Nusselt number for negative or very low inclination angles. It is found that the increase of the inclination angle from the negative to the positive ones increases the average Nusselt number. However, there is a maximum value of the average Nusselt number about 30° of inclination angle, which with a further increase of the inclination angle results in a decreasing trend of the average Nusselt number. Further, Han and Hyun [21] have dealt with the natural convection of a fluid in a parallelogrammic enclosure filled with a saturated porous medium using the Brinkman-Darcy extended flow model. The enclosure is cooled from below and heated from the top and the sidewalls are insulated. The results show that for high values of the permeability, the flow is a Darcy one.

The natural convective heat transfer of nanofluids in enclosures filled with porous media and saturated with nanofluids is a relatively new interesting area. In the present study, the mathematical proposed by nanofluid model Tiwari and Das [19] along with the new empirical correlations [22] for the heat capacitance, thermal conductivity and thermal diffusivity of the nanofluid saturated porous medium are utilized to analyze the free convective heat transfer of nanofluids in a parallelogrammic enclosures filled with nanofluid-saturated porous media when the sidewalls are subject to temperature differences. The effects of the presence of nanoparticles and porous matrix on the flow and heat transfer characteristics are examined. To the best of the authors’ knowledge, the results of the present study are new and have not been reported before.

## Basic Equations

**Fig 1:**
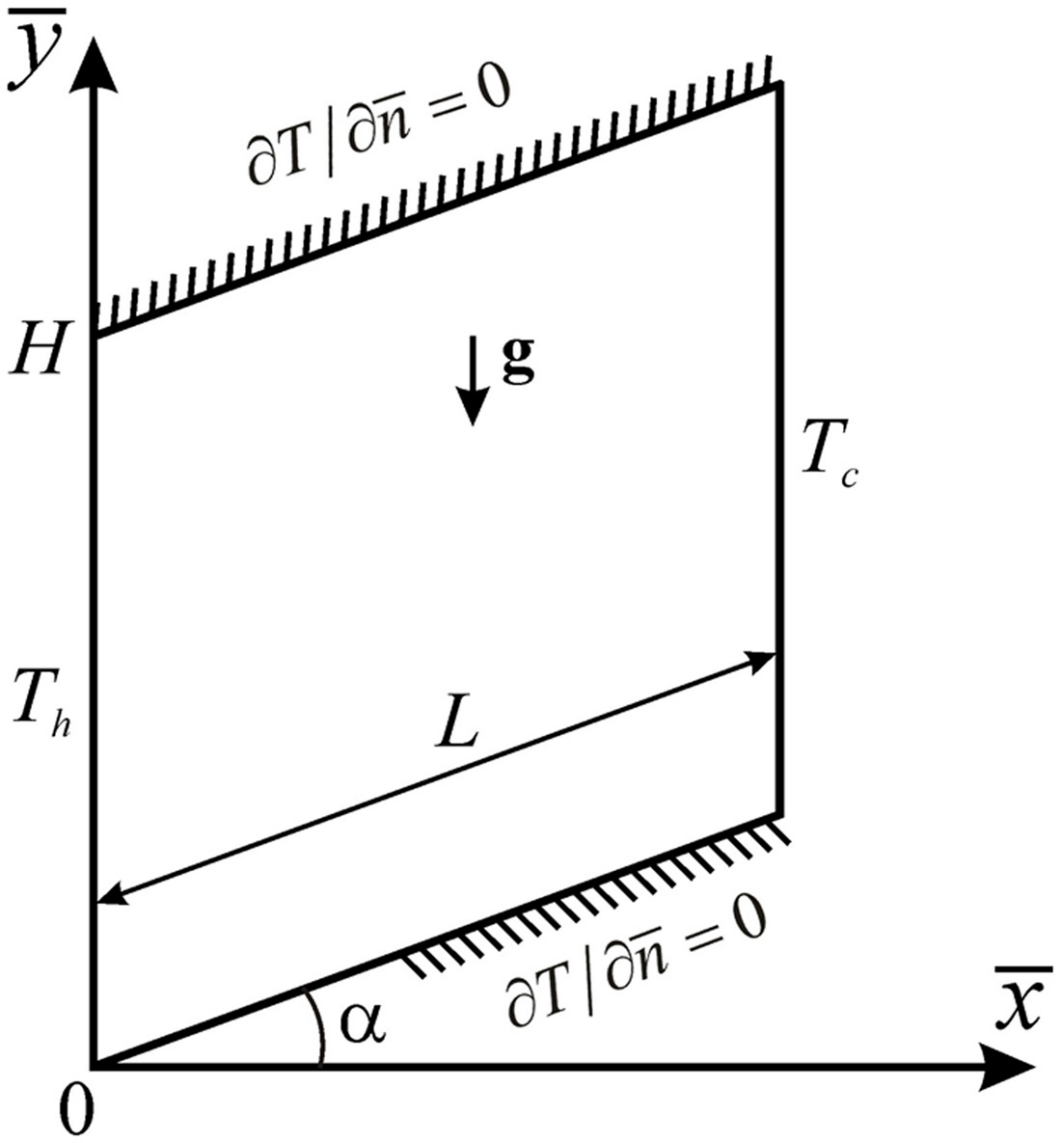
Physical model and coordinate system.

The physical situation and coordinate system are shown in Fig 1. The porous parallelogrammic cavity of height *H* and length *L* is formed by two isothermal vertical walls and two adiabatic inclined walls. Vertical walls are heated and cooled at constant temperatures *T*_*h*_ and *T*_*c*_, respectively, where *T*_*h*_ > *T*_*c*_. The cavity is filled with a Cu/water nanofluid. It is assumed, as in [23–25], that nanoparticles are suspended in the nanofluid using either surface charge technology or surfactant, which prevents the nanoparticles from deposition and agglomeration on the porous matrix. The nanofluid is considered to be Newtonian with negligible viscous dissipation and gravity acts in the negative $\overset{-}{y}$-direction. In addition, the flow is assumed to be steady state, laminar and the thermophysical properties of the nanofluid are assumed as constant, except for the density in the buoyancy term of the momentum equations, which is treated according to the Boussinesq approximation.

Further, it is considered that the temperature of the fluid phase is equal to the temperature of the solid phase everywhere in the porous layer, and the local thermal equilibrium model is valid in the present study. The porous medium is considered to be homogeneous and isotropic. All surfaces of the cavity are assumed to be impermeable to both fluid and nanoparticles. In the present investigation, the Darcy model has been adopted in the governing equations of the problem.

Taking into account the above-mentioned assumptions, the steady state governing equations for the conservation of mass, momentum and energy in the nanofluid using the nanofluid model proposed by Tiwari and Das [19] can be written as follows (Nield and Bejan [26]):

∇⋅V=0 (1)

$$0=-\nabla p-\frac{\mu_{\text{nf}}}{K}V-{\left(\rho \beta \right)}_{\text{nf}}(T-T_{0})g$$ (2)

$$\left(V\cdot \nabla )T=\frac{k_{\text{mnf}}}{{\left(\rho C_{p}\right)}_{\text{nf}}}(\frac{\partial^{2}T}{\partial {\overset{-}{x}}^{2}}+\frac{\partial^{2}T}{\partial {\overset{-}{y}}^{2}}\right)$$ (3)

where **v** is the Darcian velocity vector, *k* is the thermal conductivity, *K* is the permeability of the porous medium, *p* is the pressure, **g** is the gravitational acceleration vector, *β* is the thermal expansion coefficient, *μ* is the dynamic viscosity, *ρ* is the density, and *C*_*p*_ is the specific heat at a constant pressure.

The physical properties of the nanofluid: viscosity *μ*_nf_, thermal conductivity *k*_*nf*_ heat capacitance (*ρC*_*p*_)_nf_, and buoyancy coefficient (*ρβ*)_nf_ have been obtained using the expressions presented in [22, 27, 28]. The considered thermophysical properties of the nanofluid and solid structure of the porous medium are given in Table 1.

**Table 1:** Thermal-physical properties of fluid and Cooper (Cu) nanoparticles (see [22, 27]) and solid structure of the porous medium.

Physical properties	Glass balls	Aluminum foam	Cu	Base fluid (water)
*C*_*p*_ (J·kg^-1^·K^-1^)	840	897	385	4179
*ρ* (kg·m^-3^)	2700	2700	8933	997.1
*k* (W·m^-1^·K^-1^)	1.05	205	400	0.613
*α*×10^-7^ (m^2^·s^-1^)	4.63	846.4	1163.1	1.47
*β*×10^-5^ (K^-1^)	0.9	2.22	1.67	21

It should be noted that using relations presented in [22, 26], the physical properties of the nanofluid saturated porous medium are given by

(ρCp)mnf=ε(ρCp)nf+(1-ε)(ρCp)s=(ρCp)m[1-εφ(ρCp)f-(ρCp)p(ρCp)m],kmnf=εknf+(1-ε)ks=km{1-3εφkf(kf-kp)km[kp+2kf+φ(kf-kp)]},αmnf=kmnf(ρCp)nf (4)

where *ε* is the porosity of the porous medium, *φ* is the uniform concentration of the nanoparticles in the cavity and indices “mnf”, “s”, “m”, “nf”, “f” and “p” are related to nanofluid saturated porous medium, solid matrix of the porous medium, clear fluid saturated porous medium, nanofluid, fluid and (nano) particle.

These relations (4) can be considered as the new empirical correlations for the heat capacitance, thermal conductivity and thermal diffusivity of the nanofluid saturated porous medium due to an including correlation between not only the base fluid and solid particle properties as a wide spread situation [7, 16, 18, 19, 27, 28] but also the solid matrix of the porous medium. Such correlations between all elements of the considered system such as the base fluid, nanoparticles and solid matrix of the porous medium allow to describe the fluid flow and heat transfer in the porous system more accurately.

Eqs (1–3) can be written in Cartesian coordinates as

$$\frac{\partial \overset{-}{u}}{\partial \overset{-}{x}}+\frac{\partial \overset{-}{v}}{\partial \overset{-}{y}}=0$$ (5)

$$\frac{\mu_{\text{nf}}}{K}(\frac{\partial \overset{-}{u}}{\partial \overset{-}{y}}-\frac{\partial \overset{-}{v}}{\partial \overset{-}{x}})=-g{\left(\rho \beta \right)}_{\text{nf}}\frac{\partial T}{\partial \overset{-}{x}}$$ (6)

$$\overset{-}{u}\frac{\partial T}{\partial \overset{-}{x}}+\overset{-}{v}\frac{\partial T}{\partial \overset{-}{y}}=\alpha_{\text{mnf}}(\frac{\partial^{2}T}{\partial {\overset{-}{x}}^{2}}+\frac{\partial^{2}T}{\partial {\overset{-}{y}}^{2}})$$ (7)

The above equations can be written in terms of the stream function $\overset{-}{\psi }$ and temperature together with the following non-dimensional variables:

$$x=\bar{x}/L\text{, }y=\bar{y}/L\text{, }\psi =\bar{\psi }/\alpha_{\text{mnf}}\text{, }\theta =\left(T-T_{c}\right)/\left(T_{h}-T_{c}\right)$$ (8)

The resulting dimensionless governing equations are:

$$\frac{\partial^{2}\psi }{\partial x^{2}}+\frac{\partial^{2}\psi }{\partial y^{2}}=-Ra\cdot H(\varphi )\frac{\partial \theta }{\partial x}$$ (9)

$$\frac{\partial \psi }{\partial y}\frac{\partial \theta }{\partial x}-\frac{\partial \psi }{\partial x}\frac{\partial \theta }{\partial y}=\frac{\partial^{2}\theta }{\partial x^{2}}+\frac{\partial^{2}\theta }{\partial y^{2}}$$ (10)

with the boundary conditions

$$\begin{array}{l} \psi =0\text{, }\theta =1\text{ on }x=0\\ \psi =0\text{, }\theta =0\text{ on }x=1\\ \psi =0\text{, }\frac{\partial \theta }{\partial \bar{n}}=0\text{ on }y=x\cdot \text{tg}\left(\alpha \right)\\ \psi =0\text{, }\frac{\partial \theta }{\partial \bar{n}}=0\text{ on }y=A+x\cdot \text{tg}\left(\alpha \right) \end{array}$$ (11)

Here *Ra* = *gK*(*ρβ*)_f_(*T*_*h*_−*T*_*c*_)*L* / (*α*_m_ *μ*_f_) is the Rayleigh number for the porous medium, *α*_m_ = *k*_*m*_/(*ρC*_*p*_)_f_ is the thermal diffusivity of the clear fluid saturated porous medium, *A* = *H* / *L* is the aspect ratio, and the function *H*(*φ*) is given by

$$H(\varphi )=\frac{\left[1-\varphi +\varphi {\left(\rho \beta \right)}_{\text{p}}/{\left(\rho \beta \right)}_{\text{f}}]\;[1-\varphi +\varphi {\left(\rho C_{p}\right)}_{\text{p}}/{\left(\rho C_{p}\right)}_{\text{f}}\right]}{1-\frac{3\varepsilon \varphi k_{\text{f}}(k_{\text{f}}-k_{\text{p}})}{k_{\text{m}}[k_{\text{p}}+2k_{\text{f}}+\varphi (k_{\text{f}}\;-\;k_{\text{p}})]}}{\left(1-\varphi \right)}^{2.5}$$ (12)

and it depends on the nanoparticles concentration *φ*, as well as the physical properties of the base fluid, nanoparticles and solid structure of the porous medium.

The physical quantities of interest are the local Nusselt numbers at the left and right walls, which are defined as

$$Nu_{l}=-\frac{k_{\text{mnf}}}{k_{\text{m}}}{\left(\frac{\partial \theta }{\partial x}\right)}_{x=0}\text{, }Nu_{r}=-\frac{k_{\text{mnf}}}{k_{\text{m}}}{\left(\frac{\partial \theta }{\partial x}\right)}_{x=1}$$ (13)

and the average Nusselt numbers at the left and right walls, which are given by

$$\overset{-}{Nu_{l}}=\frac{1}{A}\int_{0}^{A}Nu_{l}\;dy;\;\overset{-}{Nu_{r}}=\frac{1}{A}\int_{0}^{A}Nu_{r}\;dy$$ (14)

## Numerical Method

The formulated boundary value problem (8)–(11) was solved numerically using the finite difference method. The used numerical algorithm was described in detail in [22, 29–32]. The developed numerical code was validated successfully (see [22]).

In the present study the results were reported for inclination angle in the range of-60°<*α* < 60°, the aspect ratio in the range of 0.1 < *A* < 10, Rayleigh number in the range of 10 < *Ra* < 1000, and the volume fraction of nanoparticles in the range of 0 < *φ* < 0.1. Results are obtained for a porous medium with porosity of *ε* = 0.9, thermal conductivity of *k*_*s*_ = 2*k*_*f*_ and for different combinations of the dimensionless parameters and different grid sizes. The results are shown in Table 2, which show that the grid size of 150×150 provides acceptable accuracy. Hence, the grid size of 150×150 is utilized for all of the computations.

**Table 2:** Grid independency for different combination of dimensionless parameters.

*α*	*A*	*Ra*	*φ*	Average Nusselt Number ${\bar{Nu}}_{l}$					
				Grid Size					
				50×50	100×100	150×150	200×200	250×250	300×300
0	1	10	0	1.0791	1.079	1.079	1.079	1.079	1.079
0	1	1000	0	13.654	13.644	13.642	13.642	13.641	13.641
0	0.1	1000	0	1.679	1.667	1.666	1.664	1.663	1.663
0	10	1000	0	5.082	5.083	5.083	5.083	5.083	5.083
-60	1	1000	0	4.283	4.300	4.303	4.304	4.304	4.304
60	1	1000	0	15.442	15.443	15.443	15.443	15.443	15.443
0	10	1000	0.1	12.467	12.464	12.463	12.463	12.463	12.463
-60	1	1000	0.1	4.068	4.078	4.080	4.080	4.080	4.080
60	1	1000	0.1	13.851	13.853	13.853	13.853	13.853	13.853

**Fig 2:**
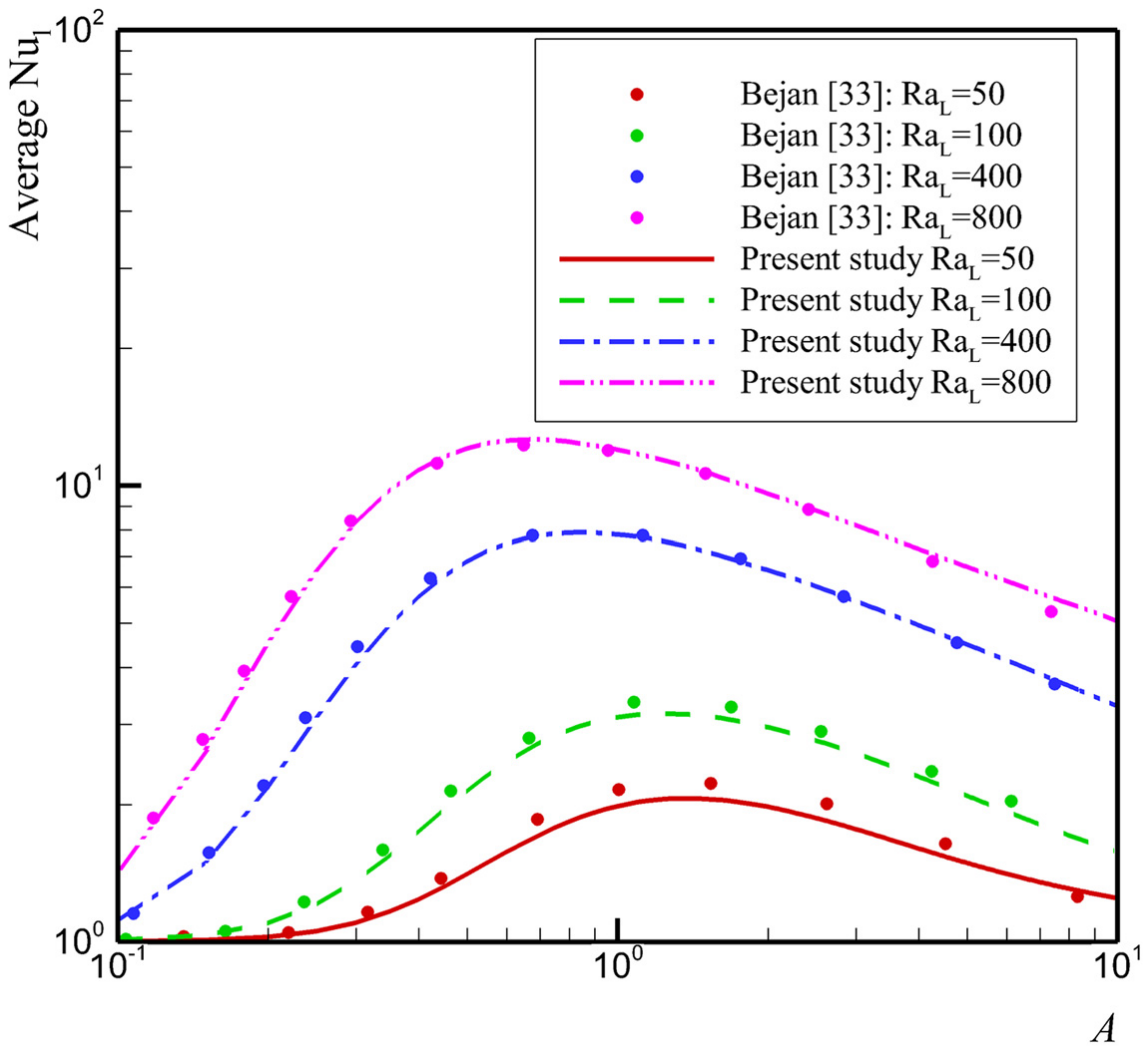
Comparison with Bejan [33] in the case of rectangular enclosure saturated with a pure fluid.

Considering zero volume fraction of nanoparticles (*φ* = 0) and for a rectangular enclosure (*α* = 0°), the present study reduces to those reported by Bejan [33]. A comparison between the present results and those by Bejan [33] is illustrated in Fig 2. As can be seen, the agreement between these results is very good. Thus, we are confident that our results are accurate and correct.

**Fig 3:**
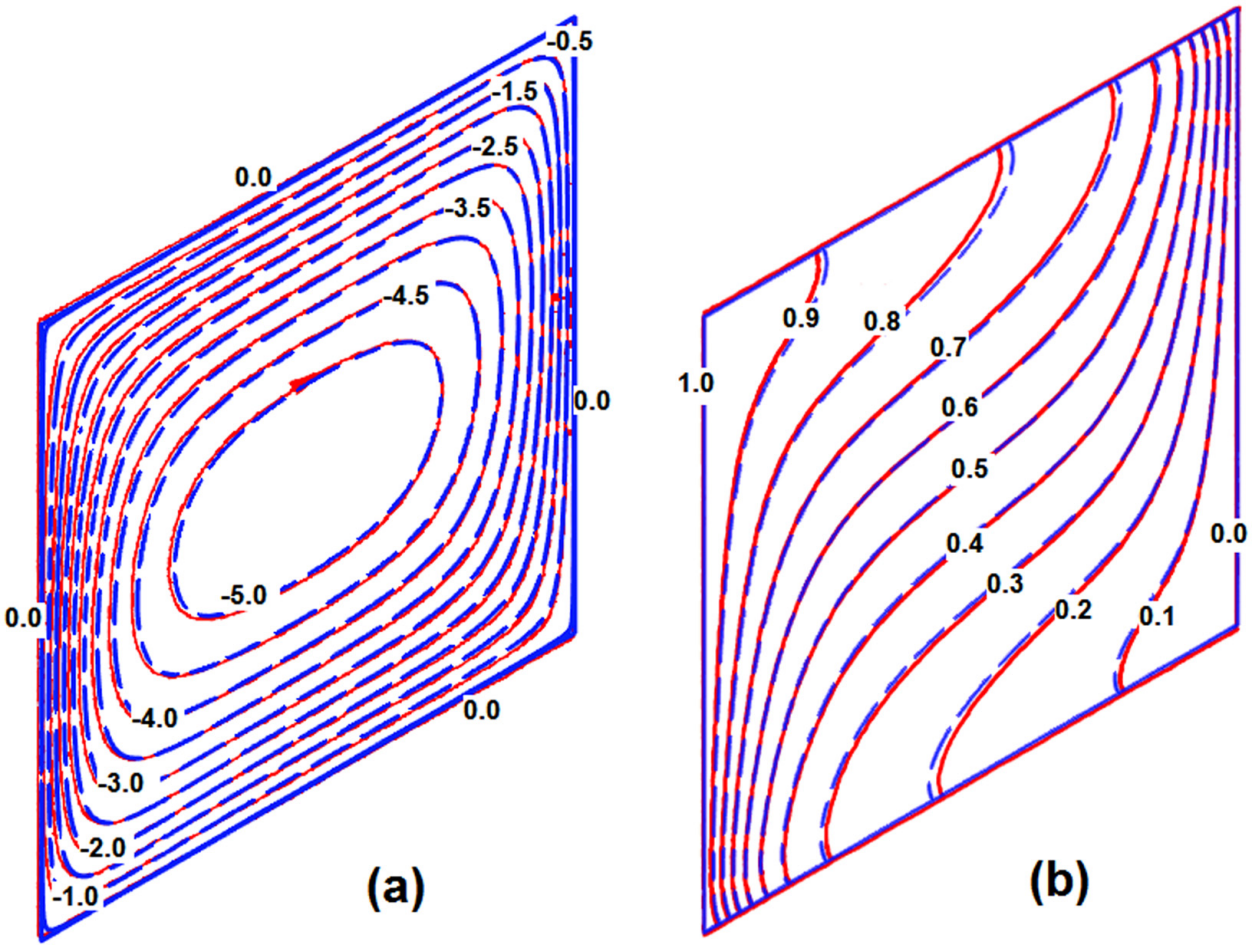
Stream lines (a) and isotherms (b) when α = 30°, A = 1 and Ra = 100: the blue with dashed lines is the present study and the solid red lines are Costa [20] (a) and (b)

**Fig 4:**
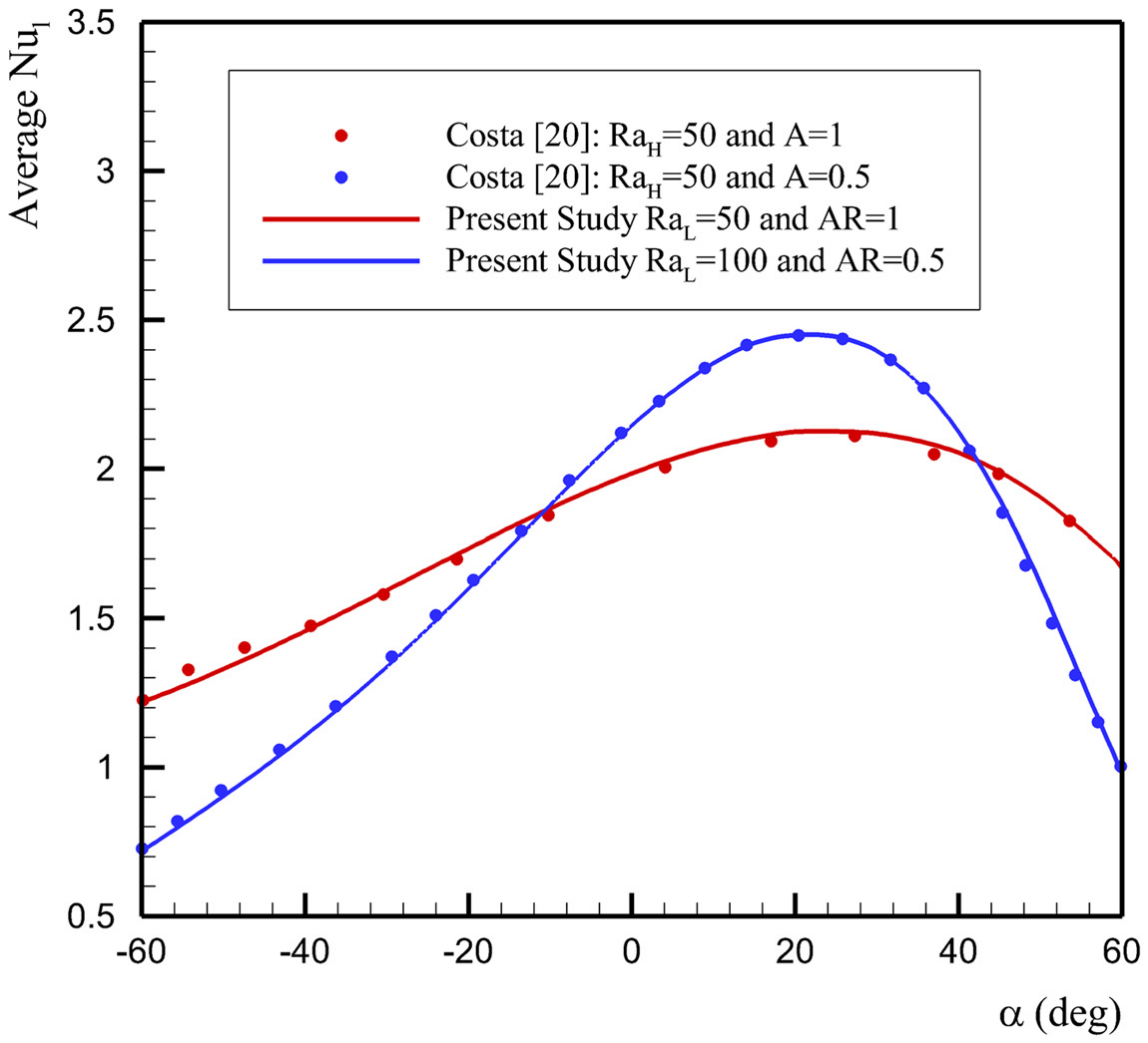
Comparison between the evaluated average Nusselt number ${\bar{Nu}}_{l}$ in the present study and those reported by Costa [20].

Further, the results of streamlines and temperature contours are compared, for *α* = 30° and *A* = 1 (square enclosure) with those reported by Costa [20] in Fig 3a and 3b, respectively. In this case, the minimum and maximum values of stream function are obtained as -5.524 and 0 in the present study, and the minimum and maximum values of stream function were reported as -5.52 and 0 in the study of Costa [20]. It is seen that there is an excellent agreement between the results of present study and the results of Costa [20]. It should be, however, mentioned that the results for the average Nusselt number calculated by Costa [20] are based on the Rayleigh number with *H* as the reference length (*Ra*_*H*_), while the results of the present study as well as those by Bejan [33] are based on the Rayleigh number with *L* as the reference length (*Ra*_*L*_). If we consider the definitions of these two Rayleigh numbers as: *Ra*_*H*_ = *gK*(*ρβ*)_f_ (*T*_*h*_−*T*_*c*_)*H*/(*α*_m_ *μ*_f_) and *Ra*_*L*_ = *gK*(*ρβ*)_f_ (*T*_*h*_−*T*_*c*_)*H*/(*α*_m_ *μ*_f_), and considering *A* = *H*/*L*, the following relation is available: *Ra*_*H*_ = *Ra*_*L*_ × *A*. In addition, in the study of Costa [20], the average Nusselt number was introduced as:$\overset{-}{Nu_{l}}=-\frac{1}{A\cdot \text{cos}(\alpha )}\int_{0}^{1}{\left(\frac{\partial \theta }{\partial x}\right)}_{x=0}dy$. Considering the definition of the Nusselt and Rayleigh numbers in the present study, the results of Costa [20] were multiplied by cos(*α*), while the results of the present study were calculated for *Ra*_*L*_ = *Ra*_*H*_/*A*. The evaluated results for the average Nusselt number are compared in Fig 4, which shows an excellent agreement between the results of present study and the results reported by Costa [20].

## Results and Discussion

A water based nanofluid, consisting of well dispersed cooper (Cu) nanoparticles, is studied. The volume fraction of nanoparticles is considered in the range of 0 < *φ* < 0.1. Two types of porous media, namely, glass balls and aluminum foam, are adopted. The thermal conductivity of glass balls is comparable with the thermal conductivity of nanofluid while the thermal conductivity of aluminum foam is comparable with the thermal conductivity of nanoparticles. Three magnitudes of porosity as *ε* = 0.3, 0.5 and *ε = 0*.*7* are adopted to show the effect of porosity. The inclination angle and aspect ratio are studied in the range of-60°< *α* < +60° and 0.1 < *A* < 10, respectively. Finally, the Rayleigh number is assumed in the range of 10 < *Ra* < 1000.

**Fig 5:**
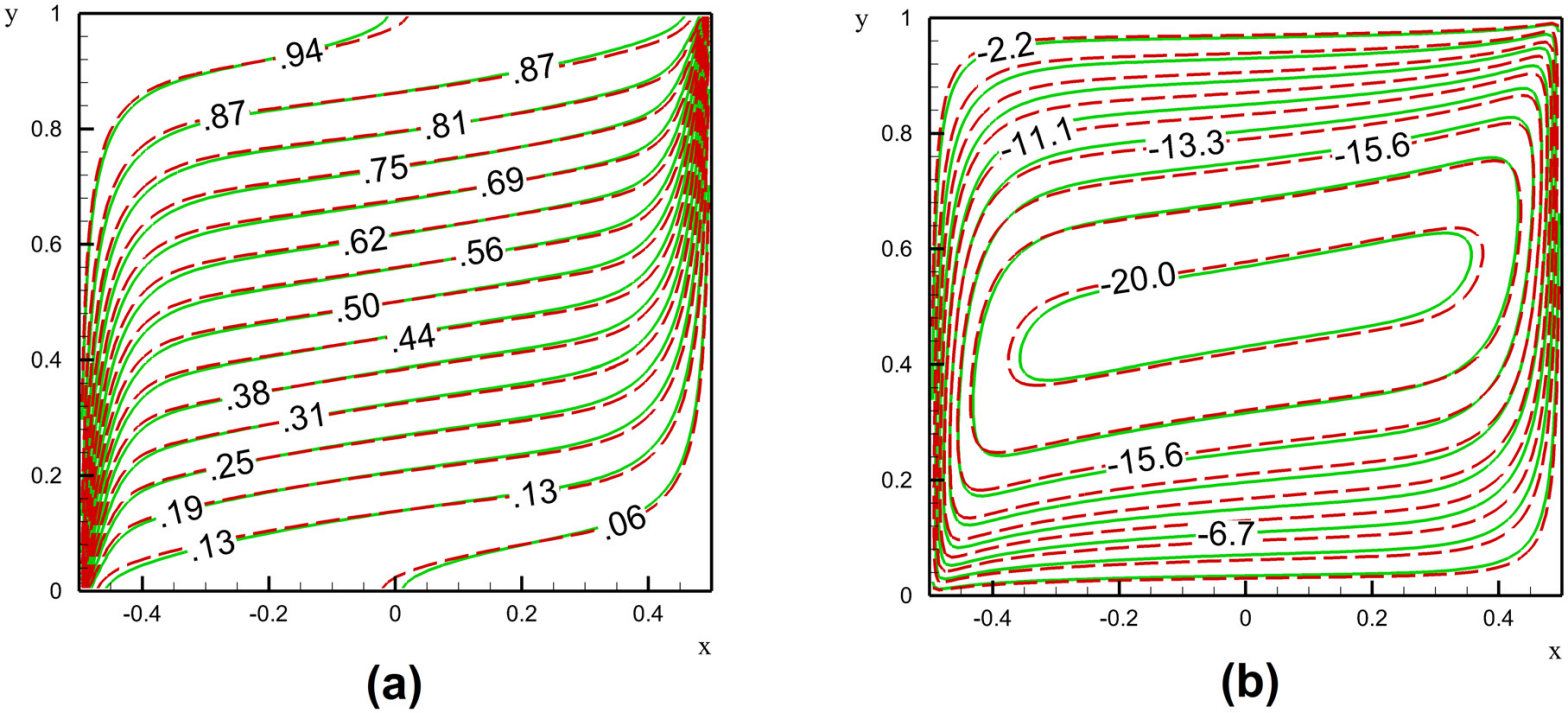
A comparison between the isotherms (a) and streamlines (b) of a nanofluid and the base fluid in a glass balls porous medium when *Ra* = 1000, A = 1, *α* = 0, *φ* = 0.05 and *ε* = 0.5; the dashed red lines are base fluid and the solid green lines are the nanofluid (a) and (b).

**Fig 6:**
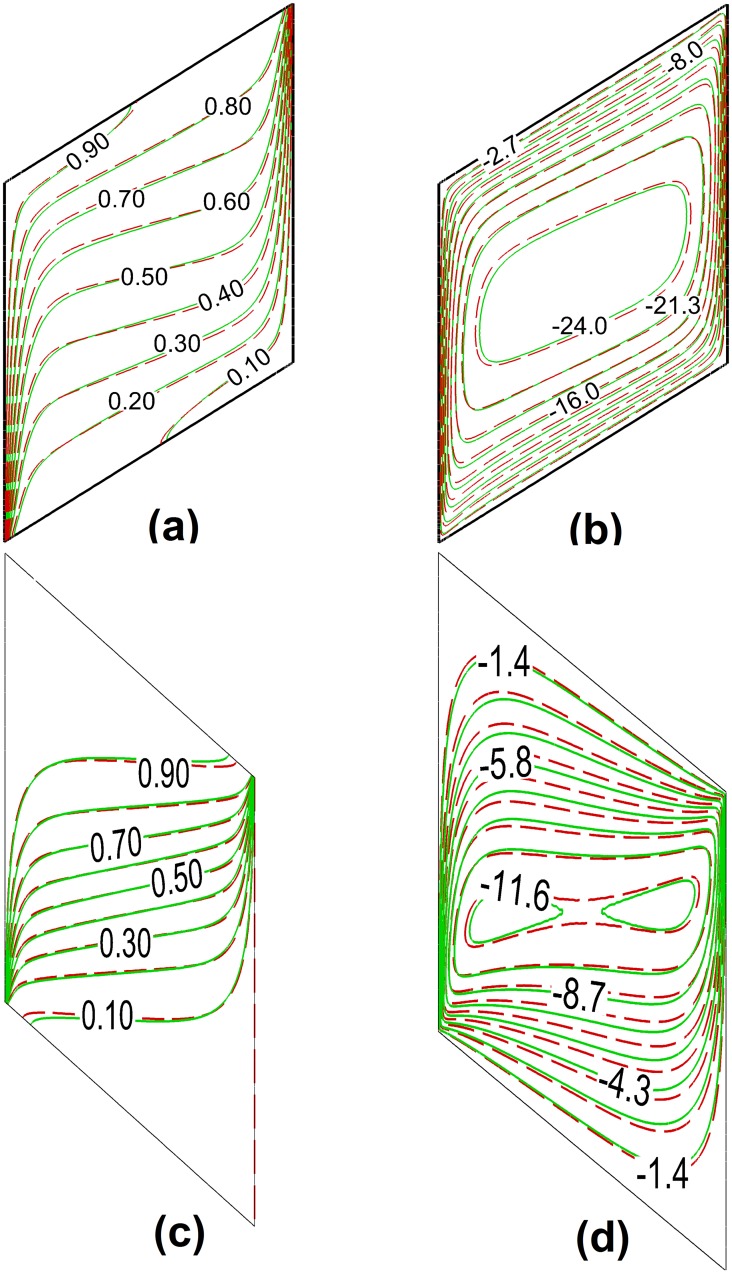
A comparison between isotherms (a), (c) and streamlines (b), (d) of the nanofluid and base fluid in a glass balls porous medium for two inclination angles of *α* = - 60° and *α* = + 60° when *Ra* = 1000, *A* = 1, *φ* = 0.05 and *ε* = 0.5; the dashed red lines are base fluid and the solid green lines are the nanofluid.

Figs 5 and 6 show the effect of presence of nanoparticles on the streamlines and isotherms for a porous square enclosure filled with glass balls and saturated with Cu-water nanofluid when *Ra* = 1000, *φ* = 0.05 and *ε* = 0.5 for different inclination angles *α*. The isotherm lines are depicted in Figs 5a, 6a and 6c; the streamlines are depicted in Figs 5b, 6b and 6d.

The minimum value of stream function for the case of nanofluid is -18.15 and for the case of base fluid is -20.5 and the maximum value in both cases is zero. The stream function in Fig 5b is in the range of -20 to 0 which is divided to 10 equally levels. Fig 6 shows the streamlines and isotherms for the positive and negatives inclination angles. As seen, in the case of positive inclination angle (i.e. *α* = +60°) the isotherms are more dispersed compared to the case of negative inclination angle (i.e. *α* = - 60°). This is because of the flow patterns.

For the case of the negative inclination angle, in the bottom-right and up-left of the cavity, there are acute angles, in which the flow tries to accelerate. When the flow stream reaches the actuate angle, it tries to avoid trapping, and hence, the streams of the main flow are squeezed in the obtuse angles. Consequently, as mentioned, the isotherms are also significantly affected by the streamline patterns. In contrast, in the case of positive inclination angles (i.e. *α* = + 60°) there are obtuse angles where the flow tries to accelerate, and hence, the flow could go inside the corners, so that a more dispersed flow pattern inside the cavity can be seen. Consequently, the isotherms are also more dispersed inside the cavity. The comparison between the isotherms of the nanofluid and the base fluid indicates that the isotherms of the nanofluid are more distributed in right angle cavities and positive inclination angle. In contrast, in the case of negative inclination angles, the isotherms of the nanofluid are less distributed. This difference is because of the effects of presence of nanoparticles on the dynamic viscosity and thermal conductivity. The results were plotted for different inclination angles of the enclosure and it is found that in the enclosure with negative inclination angles the effect of the increase of the viscosity on the flow patterns is much important than the case of the enclosure with a positive inclination angle. For the case of negative inclination angles, as the viscosity of the liquid increases (i.e. the nanofluid) the flow is more affected by the acute angles, and hence, the distribution of streamlines is less than that of the base fluid, and consequently, the isotherms are also more limited. In contrast, for the case of enclosure with positive angles the effect of viscosity on the flow patterns decreases. As the thermal conductivity of the nanofluid is higher than that of the base fluid because of the presence of nanoparticles, the isotherms of the nanofluid are more distributed than that of the base fluid.

**Fig 7:**
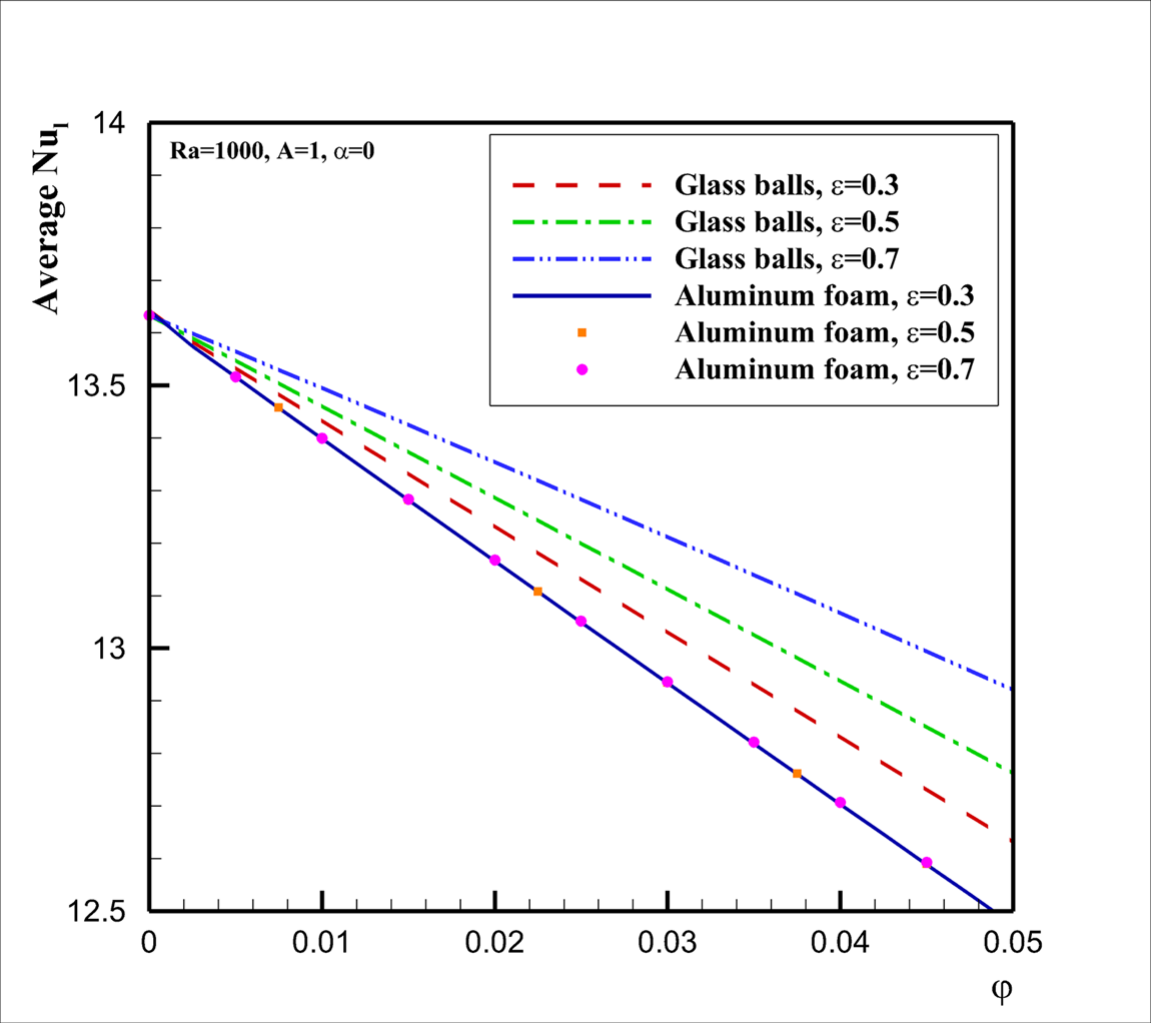
Effect of volume fraction *φ* of nanoparticles on ${\bar{Nu}}_{l}$ for different types of porous media and porosities.

Fig 7 shows the variation of the average Nusselt number ${\overset{-}{Nu}}_{l}$ with the volume fraction parameter *φ* for two different porous media of glass ball and aluminum foam. The results are reported for three different porosity of *ε* = 0.3, 0.5 and 0.7 at *Ra* = 1000, *A* = 1, *α* = 0. As seen, when the thermal conductivity of the porous matrix, compared to the thermal conductivity of the base fluid, is high (e.g. the case of aluminum foam), the variation of the porosity does not affect the heat transfer rate (i.e.${\bar{Nu}}_{l}$) inside the enclosure. This is because of the fact that the high thermal conductivity of the porous matrix is the dominant mechanism of the heat transfer and the slight variation of the thermal conductivity of the fluid because of the presence of nanoparticles could not induce a significant effect on the effective thermal conductivity of the porous medium and the fluid. In contrast, when the thermal conductivity of the porous matrix is comparable with the thermal conductivity of the base fluid (the case of glass balls), the presence of nanoparticles shows significant effect on ${\bar{Nu}}_{l}$. As the porosity of the porous media increases, the average Nusselt number also tends to raise. This is because of the fact that the increase of the porosity increases the void space (which is saturated by the fluid), and consequently, the raise of the void space boosts the effect of the presence of nanoparticles on the effective thermal conductivity. However, for the both cases of the porous media with the aluminum foam and glass balls matrixes, the presence of nanoparticles reduces the overall heat transfer rate. On the other hand, the zero volume fraction (*φ* = 0) of the nanoparticles shown in Fig 7 indicates the variation of ${\overset{-}{Nu}}_{l}$ for the base fluid. As seen, the increase of *φ* reduces the overall values of ${\overset{-}{Nu}}_{l}$. This reduction in ${\bar{Nu}}_{l}$ is because of the fact that the presence of nanoparticles boosts the viscosity of the fluid. When the viscosity increases, the velocity of the nanofluid tends to decrease, and consequently, it results in the deterioration of the heat transfer inside the enclosure. However, as mentioned, the presence of nanoparticles also increases the thermal conductivity of the fluid, which tends to increase ${\bar{Nu}}_{l}$. Therefore, Fig 7 reveals that the deterioration of the heat transfer because of the increase of the viscosity (which is the result of the presence of nanoparticles) is much more significant than the heat transfer enhancement because of the presence of nanoparticles. Moreover, the deterioration of the heat transfer by the presence of nanoparticles is more significant for the case of a porous matrix with low void fraction (a porous medium with low porosity) and a porous medium with high thermal conductivity.

**Fig 8:**
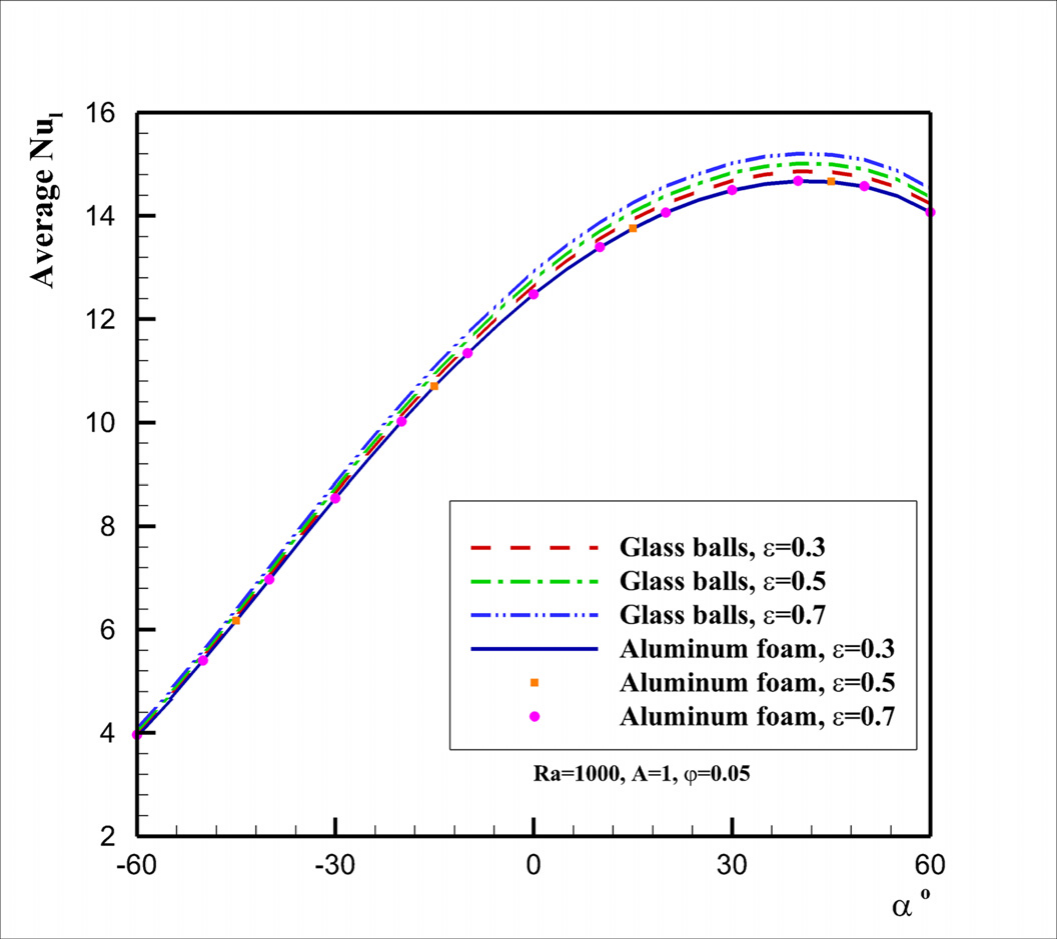
Effect of the inclination angle *α* on ${\bar{Nu}}_{l}$ for different types of porous media and porosities.

Fig 8 shows the effect of the inclination angle (*α*) on the heat transfer rate (the average Nusselt number ${\bar{Nu}}_{l}$) for two selected porous media of glass balls and aluminum foam at *Ra* = 1000, *A* = 1, *φ* = 0.05. This figure, in agreement with Fig 7, shows that the variation of porosity does not affect the variation of ${\bar{Nu}}_{l}$ for the case of porous media with aluminum foam matrix. For the case of glass balls porous matrix, Fig 8 indicates that the difference between ${\bar{Nu}}_{l}$ curves by the increase of the porosity gets more significant as the inclination angle increases from the negative angles to the positive ones. It was observed in Fig 6 that when the inclination angle is negative the flow of the fluid in the cavity is week. In this situation, the effect of augmentation of the viscosity (which is because of the presence of nanoparticles) on the flow is very strong. However, by the increase of the inclination angle the flow and velocity of the fluid inside the enclosure gets stronger, and hence, the effect of viscosity on the flow and heat transfer decreases. In this case, the enhancement of the thermal conductivity because of the presence of nanoparticles plays an important role, and the thermal conductivity enhancement boosts as the void fraction rises. It is also interesting that the increase of the inclination angle increases the heat transfer rate (${\bar{Nu}}_{l}$) and there is an optimum value of ${\bar{Nu}}_{l}$ at the inclination angle about *α* = 40°. The presence of the optimum value of inclination angle is in agreement with the results of the base fluid reported by Costa [20].

**Fig 9:**
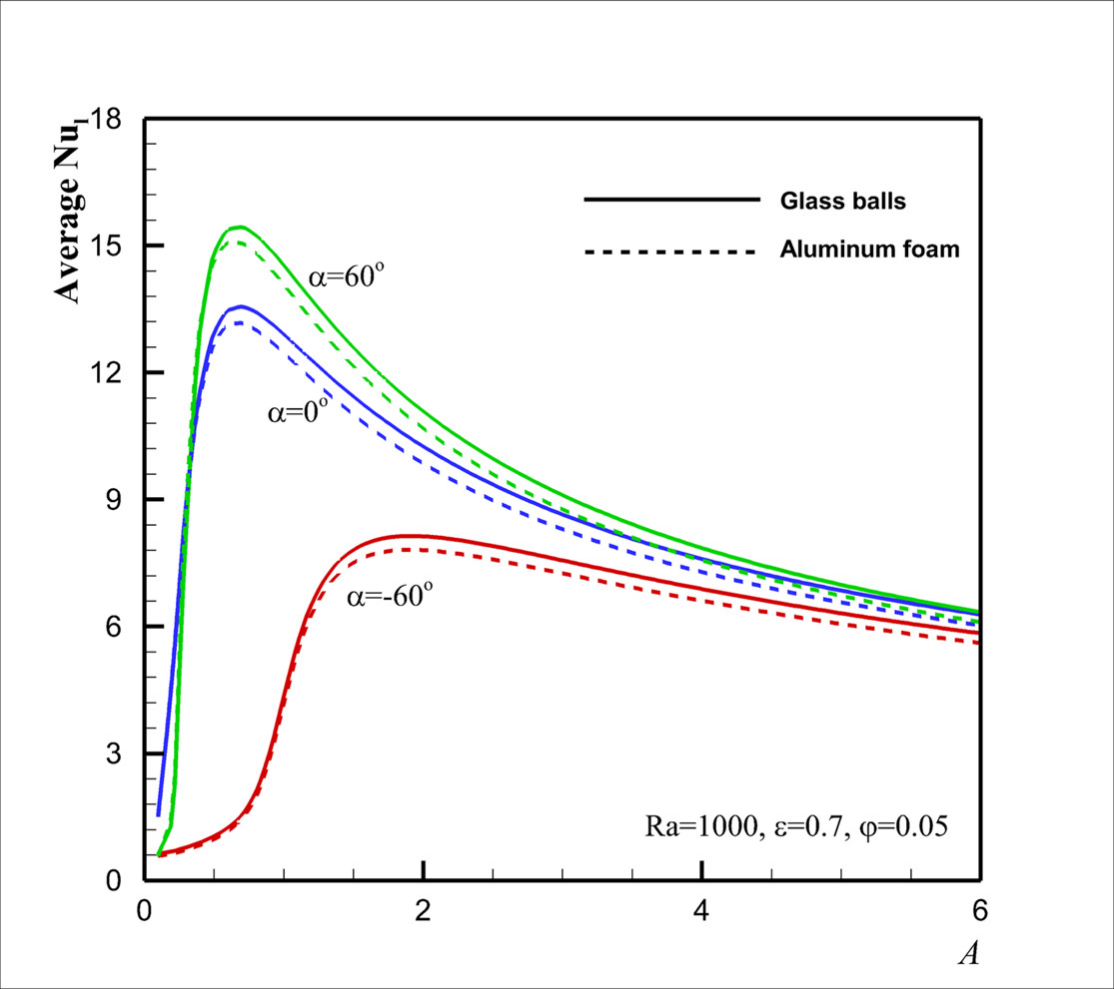
Effect of aspect ratio *A* and the porous matrix on ${\bar{Nu}}_{l}$ for selected inclination angles.

Fig 9 shows a comparison between the evaluated average Nusselt numbers ${\bar{Nu}}_{l}$ for two types of porous matrixes of aluminum foam and glass balls saturated by 5% volume fraction of Cu-water nanofluid for different aspect ratios in the range of 0.1 < *A* < 6 and three inclination angles of *α* = - 60°, *α* = 0° and *α* = + 60° at *Ra* = 1000, *ε* = 0.7, *φ* = 0.05. This figure shows, in agreement with Fig 8, that at the inclination angle of *α* = + 60°, ${\bar{Nu}}_{l}$ is higher than that of *α* = 0°, and that ${\bar{Nu}}_{l}$ for the porous matrix of the glass balls is also higher than that of the aluminum foam.

Fig 9 shows that for very small aspect ratios the average Nusselt number is very small. The small aspect ratio (*A* ≈ 0.2) indicates a wide enclosure in which the heat transfer is dominated by the conduction regime and temperature varies almost linearly across the enclosure (Regime I). However, because of the horizontal temperature gradient, there is a slow clockwise circulation. The slow circulation flow can carry small amount of heat among the adiabatic horizontal walls in top and bottom of the enclosure. Indeed, the effect of this circulation flow on the heat transfer inside the enclosure is not significant. The increase of the aspect ratio (0.1 < *A* < 1) tends to break the liner temperature distribution (break regime I) across the cavity and induce a natural convective heat transfer with a strong natural circulation regime across the enclosure (Regime II). In this case, the increase of the aspect ratio (*A*) would significantly increase the heat transfer. For an enclosure with large values of the aspect ratio (a tall enclosure), vertical boundary layers are formed distinctly along the vertical hot and cold walls. The top adiabatic wall is lined with a distinct hot thermal layer, and the bottom one is lined with a distinct cold thermal layer. The core part of the enclosure would remain relatively stagnant and thermally stratified (Regime III). The isotherms and streamlines for this case can be seen in Fig 5. Hence, as the aspect ratio increases (*A* >> 1) the heat transfer tends to decrease. Therefore, as seen in Fig 9, there is an optimum value of the aspect ratio, in which the average Nusselt number is maximum. For an enclosure with positive inclination angles, the conductive dominant regime (Regime I) can be break in smaller aspect ratios as the fluid could more freely flow in an obtuse angle. For the enclosures with negative inclination angles, the breakdown of regime I would be postponed to higher aspect ratios as the fluid could not freely flow in acute angles. Fig 9 also indicates that there is no significant difference between the constricting porous matrixes when the aspect ratio *A* of the enclosure is very low. However, as *A* reaches to unity and higher values, a significant difference between ${\bar{Nu}}_{l}$ numbers, evaluated for the selected porous matrixes, can be seen. This is because of the fact that the augmentation of the fluid viscosity (because of the presence of nanoparticles) is the dominant effect in the enclosures with medium and high aspect ratios (*A* > 1). As the aspect ratio *A* increases, the buoyancy induced flow inside the enclosure gets stronger, and consequently, the viscosity effect reduces. For this case, the thermal conductivity of the nanofluid also plays a significant role and induces significant differences between the evaluated values of ${\bar{Nu}}_{l}$.

**Fig 10:**
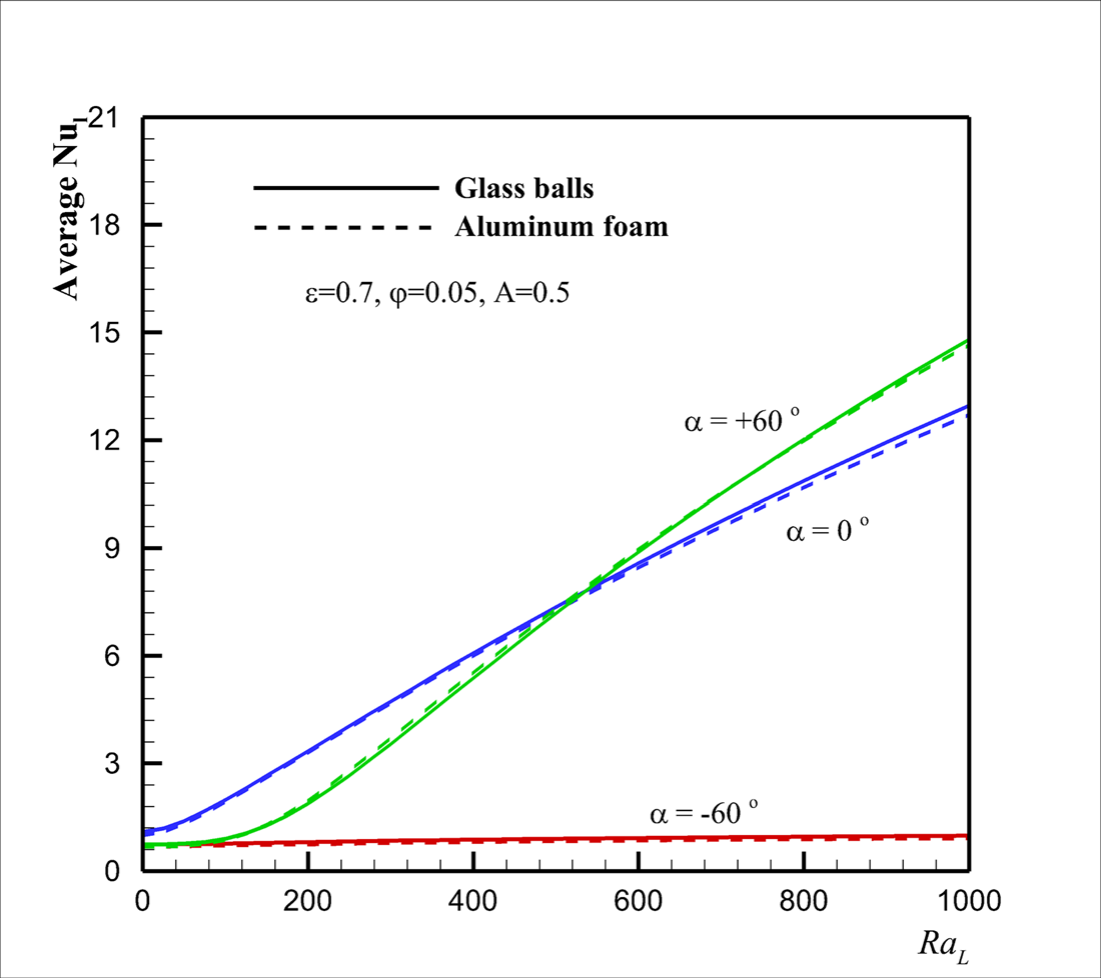
Effect of *Ra* and *α* on ${\bar{Nu}}_{l}$ for the two selected porous matrixes in the case *A* = 0.5.

**Fig 11:**
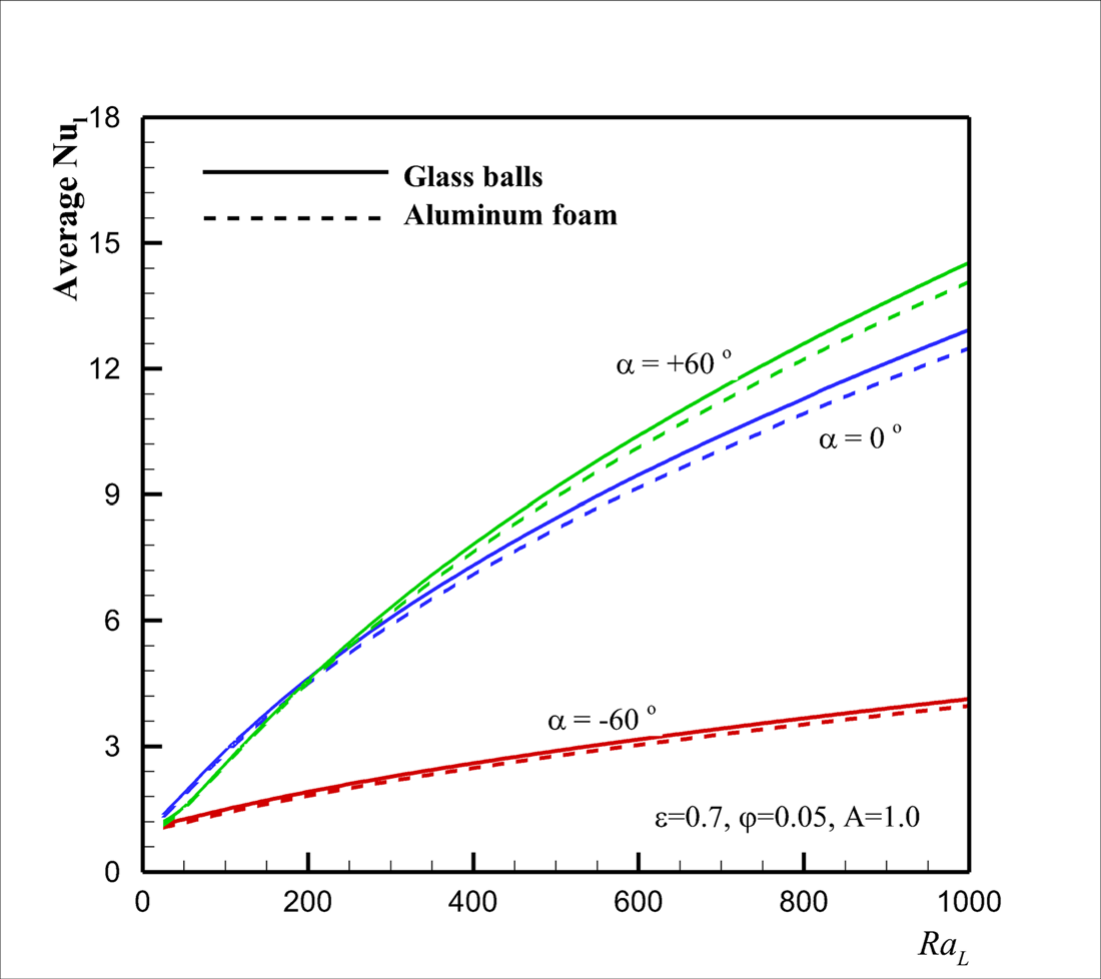
Effect of *Ra* and *α* on ${\bar{Nu}}_{l}$ for the two selected porous matrixes in the case *A* = 1 (square cavity).

**Fig 12:**
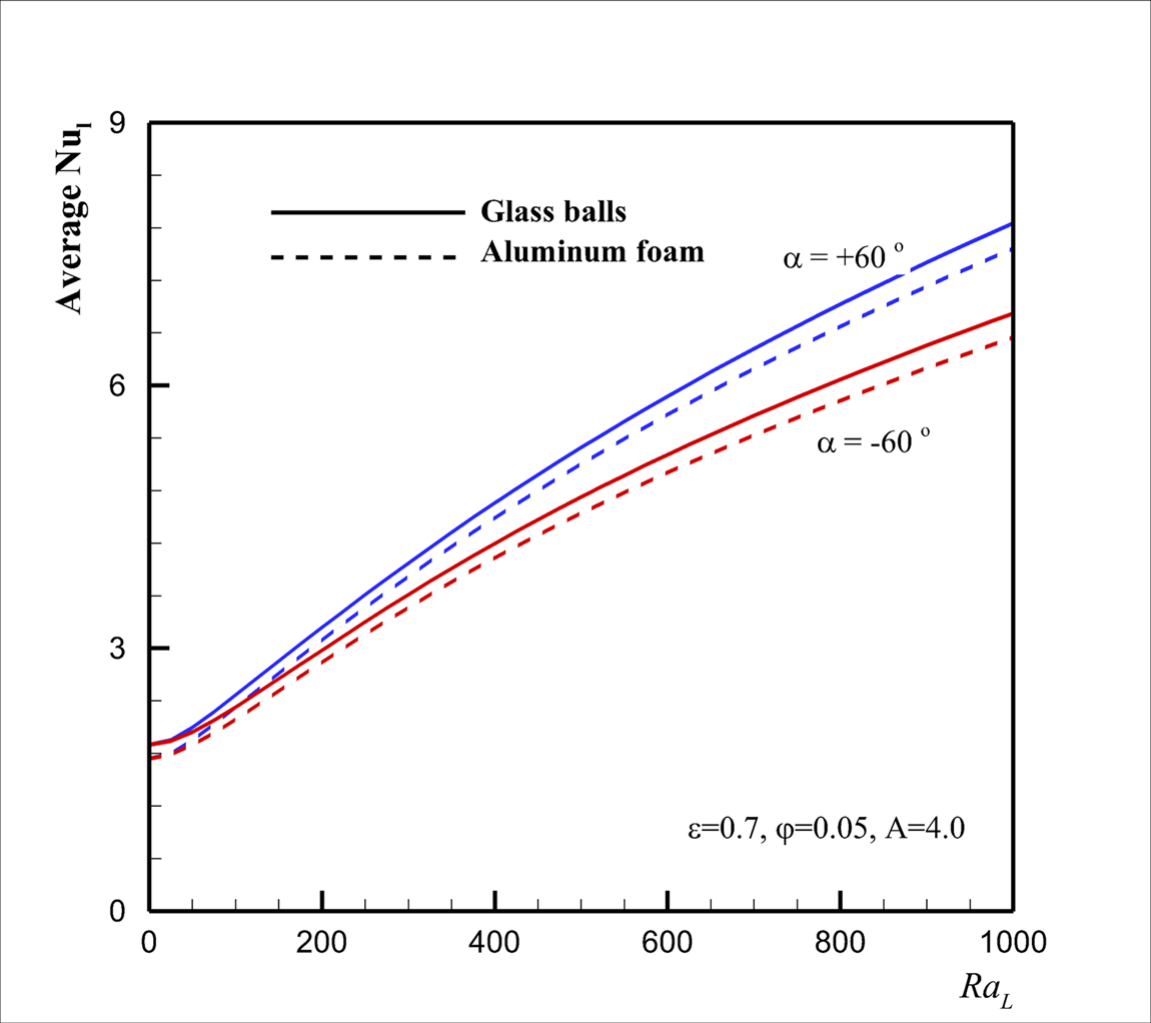
Effect of *Ra* and *α* on ${\bar{Nu}}_{l}$ for the two selected porous matrixes in the case *A* = 4.0.

Finally, Figs 10, 11 and 12 show the effect of the Rayleigh number *Ra* on ${\bar{Nu}}_{l}$ in case of two types of porous matrixes of aluminum foam and glass balls for the selected aspect ratios *A* of 0.5, 1 and 4, respectively, for three inclination angles of *α* = - 60°, *α* = 0° and *α* = + 60° at *ε* = 0.7, *φ* = 0.05. The results are plotted for two selected porous matrixes and different values of inclination angles. These figures show that for high values of *Ra* (*Ra* ≈ 1000), in agreement with Fig 9, ${\bar{Nu}}_{l}$ for the porous matrix of glass balls is higher than that for the aluminum foam. However, when the aspect ratio is lower than unity and the inclination angle is positive and large, the porous matrix with the aluminum foam could induce a slightly higher value of ${\bar{Nu}}_{l}$ rather than the case of porous with glass balls. For moderate values of *Ra* and low values of *A*, both of the thermal enhancement and the viscosity augmentation effects (which are the result of the presence of nanoparticles) are significant and could result to the slight variation of ${\bar{Nu}}_{l}$ for two cases of porous matrixes of glass balls and aluminum foam. When the inclination angle and aspect ratio are very low (i.e. *α* = - 60° and *A* = 0.5), Fig 10 indicates that the increase of *Ra* does not alter the variation of ${\bar{Nu}}_{l}$. In this case, the heat transfer is mostly because of the conduction rather than the flow stream. When the inclination angle increases to the positive values, the increase of the *Ra* boosts the flow strength inside the enclosure and the heat transfer rate (${\bar{Nu}}_{l}$) increases. For *Ra* below *Ra* = 500 the values of ${\bar{Nu}}_{l}$ for a parallelogrammic enclosure with right angles *α* = 0° is higher than that of *α* = + 60°. When *Ra* reaches to values about 500 and higher the heat transfer for the parallelogrammic enclosure with *α* = + 60° is higher than that of *α* = 0°. Figs 11 and 12 show that this phenomenon occurs at a lower values of *Ra* (i.e. *Ra* ≈ 200) as *A* (aspect ratio) raises (i.e. *A* = 1.0), and it could be suspended for a very large value of *A* (i.e. *A* = 4.0). These observations are the direct result of the flow patterns and interaction of flow with acute and obtuse angles. Comparison between Figs 10 and 11 indicates that the augmentation of the aspect ratio induces a positive effect on the heat transfer for the negative inclination angle and results in the increase of heat transfer. For this case (i.e. *α* = - 60° and *A* = 1.0), the raise of *Ra* induces a slight free convection flow stream inside the enclosure which results in slight augmentation of heat transfer rate. For negative inclination angles, Fig 12 confirms that the further increase of the aspect ratio (*A* = 4.0) increases the positive heat transfer effects, and consequently, the increase of *Ra* significantly increases the values of ${\bar{Nu}}_{l}$. Figs 11 and 12 also suggest that the values of ${\bar{Nu}}_{l}$ are always higher than that of the aluminum foam for a porous matrix of the glass balls. This is because of the fact that the thermal conductivity of the glass balls is low, and hence, the presence of the highly conductive copper nanoparticles in the fluid can significantly affect the effective thermal conductivity of the porous medium and the nanofluid. For the case of aluminum foam, the thermal conductivity of the porous matrix is very high, and hence, the presence of a low volume fraction of conductive nanoparticles in the host fluid cannot induce a significant effect on the effective thermal conductivity of the porous medium and the nanofluid. Thus, the values of ${\bar{Nu}}_{l}$ for the case of glass balls are higher than that of the corresponding aluminum foam.

## Conclusion

The heat transfer of nanofluids inside a porous parallelogrammic enclosure filled with a nanofluid is numerically studied in this paper. To solve such a problem the mathematical nanofluid model proposed by Tiwari and Das [19] has been used. The effect of the governing parameters, such as porous matrix, volume fraction of nanoparticles, the inclination angles and the aspect ratio on the flow and heat transfer characteristics are analyzed. The results show that the presence of nanoparticles deteriorates the heat transfer in all studied cases. This deterioration is mostly because of the augmentation of the dynamic viscosity by the presence of nanoparticles. Therefore, it can be concluded that the nanofluids are not sufficient for heat transfer applications in porous media. In addition, in applications in which the nanoparticles are dispersed in the base fluid for their advantages such as antibacterial properties or increase of dielectric properties, the analysis of heat transfer of the utilized nanofluid is essential to avoid system over heat or system failure because of reduction of heat transfer. It is also found that the decrease of the porosity increases the porous matrix thermal conductivity while the decrease of the inclination angle and of the aspect ratio would boost the deterioration of heat transfer.
